# Proceedings of the Æsculapian Society

**Published:** 1855-07

**Authors:** T. D. Washburn, F. R. Payne

**Affiliations:** President; Secretary


### MEDICAL NEWS.

*Proceedings of the Æsculapian Society.*

The Æsculapian Society met at the 1st. Congregational Church in Marshall, Ill., on Wednesday, the 16th day of June, 1855, Dr. Washburn in the Chair. About forty members attended the meeting. One of the censors being absent, Dr. Thompson was appointed to fill the vacancy.

The reading of essays being in order, Dr. F. R. Payne read a paper on the Medicinal properties and use of Iodine, which was discussed at some length. Dr. Eaton being present was on motion invited to take a seat with the members of the society. The report of the censors being favorable, the following gentlemen were unanimously elected members of the society: Drs. W. D. Craig and J. R. Willet, of Georgetown, Ill.; Dr. W. Leachman, of York, Ill.; and O. Q. Herrick, of Midway, Ill. On Motion of Dr. F. R. Payne—

*Resolved*, That to become a member of this society, it will be required of each applicant to read and defend a medical thesis before the society, and pass a satisfactory examination before the board of censors. The society then adjourned to meet at half-past one o'clock P. M. Evening Session.

The committee appointed to revise the Constitution, By-Laws, and Code of Ethics, reported through their Chairman, Dr. York. After some amendments the report was adopted, and one thousand copies ordered to be printed, on motion of Dr. S. Thompson.

*Resolved*, That as the Constitution, By-Laws, Code of Ethics, published and printed in 1844, have been corrected and revised, and the new draught of the Constitution, By-Laws, and Code of Ethics of the National Medical Association now adopted, shall take the place of the old printed form; but this shall not abrogate any By-Laws or Resolutions on the record of this society.

D. J. D. Mitchel reported a case of complicated labor, with shoulder presentation.

Dr. York reported some interesting cases orally. Dr. Stormant read an able paper on the nature and treatment of Eclampsia.

The society adjourned to meet at half-past seven o'clock P. M.

EVENING SESSION.

At night the society met. A respectful audience had assembled to hear a public address. Dr. Richmond was introduced and entertained the company with a very able and interesting address on the rise and progress of medical science. He urged the importance of legalizing dissections, and post mortem examinations.—After the address the society continued in session.

On motion of Dr. Stormant—

*Resolved*, That the thanks of this society are hereby tendered to Dr. Richmond for his able address, and that he be requested to furnish a copy of the same to the committee of publication for the use of the society.

Dr. Richmond read an essay on Morbus Coxarius. The society adjourned to meet at 8 o'clock to-morrow morning.

MORNING SESSION.

The society met, and on motion of Dr. Logan, Drs. Hamilton and Washburn were added to the publishing committee.

On motion of Dr. Richmond—

*Resolved*, That a committee of three be appointed to draft a bill to legalize dissections in this State, and secure to physicians the privilege of importing subjects and dissecting materials from any commercial place where they can be legally obtained, and report to this society at its next meeting, whereupon the Chair appointed Drs. Richmond, Banks and Stormant said committee.

Dr. Hunt read a paper on the use of Calomel in which several cases were reported.

Dr. Washburn reported a case of puerperal convulsions, Dr. Hamilton reported a case of inversion of uterus. Dr. McClure presented some very important cases orally. Dr. McCord read an essay on "pneumonia," which elicited a very interesting practical discussion.

On motion of Dr. F. R. Payne, Dr. S. W. Thompson was appointed to attend the State Medical Society.

On motion of Dr. McCord—

*Resolved*, That we, the members of this society, are in favor of the prohibitory liquor bill as presented to the voters of this state, believing that if it is adopted by the people, it will add to their health, longevity and morals. After an animated debate the resolutions passed. Society then adjourned to meet at half-past one o'clock P. M.

EVENING SESSION.

The following gentlemen were appointed to read essays at the next meeting. Drs. J. Ten Brook, W, D. Craig, L. C. Churchill, A. W. McClure, H. W. Davis, H. R. Payne, and Maxwell.

On motion, Dr. D. W. Stormant was appointed to deliver the next public address.

On motion of Dr. McClure—

*Resolved*, That we will patronize as far as possible those druggists who do not engage in the sale of patent medicines.

On motion of Dr. Hamilton—

*Resolved*, That the thanks of this socity are tendered to the trustees of the Congregational Church for the use of their house during the session of the society.

On motion—

The Secretary was instructed to forward a copy of the proceedings of this meeting to the Editors of Paris and Marshall papers, Greenup Tribune, North-Western Medical Journal, and Vincennes Gazette, and that they be requested to publish the same.

On motion, the Society adjourned to meet at Charleston on the last Wednesday in November next.

T. D. WASHBURN, M. D., President.

F. R. PAYNE, M. D., Secretary.

